# Delays in antiretroviral therapy initiation among HIV-positive individuals: results of the positive living with HIV study

**DOI:** 10.3402/gha.v9.31550

**Published:** 2016-06-30

**Authors:** Krishna C. Poudel, David R. Buchanan, Kalpana Poudel-Tandukar

**Affiliations:** 1 Department of Health Promotion and Policy, School of Public Health and Health Sciences, University of Massachusetts Amherst, Amherst, MA, USA; 2 College of Nursing, University of Massachusetts Amherst, Amherst, MA, USA

**Keywords:** antiretroviral therapy, CD4+ cell count, family support, HIV/AIDS, Nepal

## Abstract

**Background:**

Lack of early initiation of antiretroviral therapy (ART) remains a major health concern due to increased risk of premature mortality and further HIV transmission. This study explored CD4+ cell count monitoring in relation to delays in ART initiation among HIV-positive individuals in the Kathmandu Valley, Nepal, where ART coverage was only 23.7% in 2011.

**Design:**

We recruited a total of 87 ART-naïve, HIV-positive individuals aged 18 to 60 years through the networks of five non-government organizations working with HIV-positive individuals. We collected data on the history of ART initiation, CD4+ cell count monitoring, socio-demographic variables, perceived family support (measured with 10-item Nepali Family Support and Difficulty Scale), depression, and HIV symptom burden. Correlates of ART eligibility were examined using multivariable logistic regression analysis.

**Results:**

A total of 72 of the 87 ART-naïve participants (82.8%) had monitored their CD4+ cell count in the past 6 months. Of these, 36 (50%) participants were eligible for ART initiation with CD4+ cell count <350 cells/mm^3^. A total of 12 participants had CD4+ cell count <200 cells/mm^3^. Lower level of perceived family support was associated with 6.05-fold higher odds (95% confidence interval =1.95 to 18.73) of being ART eligible with a CD4+ cell count <350 cells/mm^3^.

**Conclusions:**

High rate of delays in ART initiation and the strong association of low perceived family support with ART eligibility in our study participants suggest that HIV service providers should consider the role and impact of family support in influencing individual decisions to initiate ART among eligible HIV-positive individuals.

## Introduction

Antiretroviral therapy (ART) has dramatically reduced HIV-related morbidity and mortality among HIV-positive individuals (1). Although the World Health Organization (WHO) has recently recommended initiation of ART in everyone living with HIV (2), ART coverage at the end of 2013 was only 38% for adults and 24% for children globally (3). The number of people receiving ART has been rapidly increasing in the recent years (9.7 million in 2012 to 15.8 million as of June 2015, for example) (4, 5). However, high rates of late ART initiation (6–8) remain a significant challenge in improving treatment outcomes due to increased risk of premature mortality and further HIV transmission (6–9).

After HIV diagnosis, bringing eligible individuals into treatment is an important step for timely ART initiation. Studies have reported the results of CD4+ cell count at ART initiation as well as factors associated with late ART initiation or early mortality after ART initiation (6–8). These studies show that many HIV-positive individuals fail to access regular care after being diagnosed and/or obtaining CD4+ cell count results (10). Few studies to date have examined CD4+ cell count monitoring and delays in ART initiation among HIV-positive individuals outside of the clinical settings.

This study explored CD4+ cell count monitoring, delays in ART initiation, and factors associated with ART eligibility among HIV-positive individuals recruited through a network of five non-government organizations (NGOs) working with HIV-positive individuals in the Kathmandu Valley, Nepal, a resource-limited country in South Asia, where ART coverage in 2011 was only 23.7% (11).

## Methods

This study is based on data from a longitudinal study entitled ‘positive living with HIV’ (POLH) among HIV-positive individuals in the Kathmandu Valley (12–15). The baseline survey of the POLH study was conducted in February–March 2010 among 322 HIV-positive individuals aged 18–60 years, who self-reported their HIV-positive diagnosis, provided written informed consent to participate in the study voluntarily, and resided in the Kathmandu Valley, where 7 of the country's 42 ART sites are located. In Nepal, free ART services were introduced in 2004. HIV-positive individuals with CD4+ cell count <350 cells/mm^3^ (or in WHO Stage 3 or 4, irrespective of CD4+ cell count) were eligible for ART in the country during the study period (16). HIV-positive individuals who meet these criteria receive counseling on treatment initiation from their service providers. Before treatment initiation, they are entitled to the evaluation of several health outcomes, including hepatitis and tuberculosis. Those who do not return to the clinic for the evaluation of these health outcomes are likely to stay without treatment, as the monitoring of attrition in pre-ART care is generally underdeveloped in the country (17).

For the purposes of this study, we selected all of the 87 ART-naïve, HIV-positive individuals found in the POLH study baseline survey. We collected information on socio-demographic parameters, substance use, alcohol use, smoking, depression, internalized stigma, and HIV-related clinical and treatment factors through face-to-face interviews. A questionnaire was developed based on previous studies conducted in Nepal (18–20).

Perceived family support was measured with a 10-item Nepali Family Support and Difficulty Scale (*α*=0.81) specifically developed for use in Nepal (21). Each item was measured on a 4-point scale (0 ‘Not at all’ to 3 ‘All the time’). The total score of perceived family support was obtained by summing all items (after reverse-scoring negatively formulated items). With a range of 0–30, lower scores indicated lower levels of perceived family support. We measured HIV symptom burden in the past month using a 16-item index (*α*=0.92) (22). We assessed depressive symptoms over the past 2 weeks using the validated Nepali version of the 21-item Beck Depression Inventory-I (*α*=0.88) (23).

Participants were asked if they had monitored their CD4+ cell count in the past 6 months. We asked those who had monitored their CD4+ cell count to provide us with a copy of their laboratory test results from the national public health laboratory.

For data analysis, first, we compared participants who monitored their CD4+ cell count with those who did not. Second, we examined bivariate associations of each independent variable with ART eligibility (having CD4+ cell count of <350 cells/mm^3^ in the past 6 months). Finally, using multivariable logistic regression analysis, we explored the correlates of ART eligibility, including all the variables associated with the outcome variable at *p*<0.20 in the bivariate analysis as suggested by Katz (24). We used SPSS Statistics 22.0 (SPSS Inc., Chicago, USA) to perform all analyses.

## Results

The mean age of the 87 ART-naïve participants was 31.8 (SD=6.3) years; 71% were men, 62% were currently married, and 62% were employed (Table 1). Seventy-two (83%; all the females and 76% of the males) had monitored their CD4+ cell count in the past 6 months. Twelve participants had a CD4+ cell count <200 cells/mm^3^ and 36 were ART eligible due to having a CD4+ cell count <350 cells/mm^3^. Approximately one-fifth of the participants reported using illicit drugs in the past 6 months and one-fifth suffered from depressive symptoms. A significantly higher proportion of the participants who did not monitor their CD4+ cell count reported current smoking and a history of using illicit drugs. A significantly higher proportion of male participants reported current smoking (82.3% vs. 16.0%; *p*<0.001) and a history of using illicit drugs (32.3% vs. 0.0%; *p*=0.003) than female participants.

**Table 1:** Characteristics of participants according to the availability of their CD4+ cell count results (*n*=87)

	Availability of CD4+ cell count results		
		
	Yes (*n*=72)	No (*n*=15)	
Variable	*n* (%)	*n* (%)	*p*
Age (years)			
20–30	33 (78.6)	9 (21.4)	
31–47	39 (86.7)	6 (13.3)	0.318
Sex			
Female	25 (100.0)	0 (0.0)	
Male	47 (75.8)	15 (24.2)	0.007
Current marital status			
Single	28 (84.8)	5 (15.2)	
Married	44 (81.5)	10 (18.5)	0.687
Education			
Up to primary	20 (87.0)	3 (13.0)	
Secondary or higher	52 (81.3)	12 (18.8)	0.534
Employed			
No	23 (79.3)	6 (20.7)	
Yes	49 (84.5)	9 (15.5)	0.547
Months since testing HIV positive (Median=53.0)			
1–53	35 (79.5)	9 (20.5)	
54+	37 (86.0)	6 (14.0)	0.368
HIV disclosure to any family member^a^			
No	11 (68.8)	5 (31.3)	
Yes	60 (85.7)	10 (14.3)	0.107
Illicit drug use, past 6 months			
No	60 (89.6)	7 (10.4)	
Yes	12 (60.0)	8 (40.0)	0.002
Current smoker			
No	31 (96.9)	1 (3.1)	
Yes	41 (74.5)	14 (25.5)	0.008
Alcohol use, past 30 days			
No	57 (85.1)	10 (14.9)	
Yes	15 (75.0)	5 (25.0)	0.295
HIV symptom burden^b^ (Median=30.5)			
Low (16–30)	36 (83.7)	7 (16.3)	
High (31–74)	36 (81.8)	8 (18.2)	0.814
Depressive symptom burden			
No (BDI-I<20)	58 (81.7)	13 (18.3)	
Yes (BDI-I≥20)	14 (87.5)	2 (12.5)	0.578
History of any disease, past 12 months			
No	27 (79.4)	7 (20.6)	
Yes	45 (84.9)	8 (15.1)	0.508
Internalized stigma score^c^ (Median:11)			
Low (7–10)	33 (80.5)	8 (19.5)	
High (11–14)	39 (84.8)	7 (15.2)	0.597

a One participant did not respond to this question.

b Symptoms included fatigue, fever, dizziness, hand/foot pain, memory loss, nausea or vomiting, diarrhea, skin problems, cough, headache, appetite loss, bloating, muscle/joint pain, fat deposit or weight gain, weight loss, and hair loss.

c We measured internalized stigma using 7-item scale (*α*=0.72) (e.g. ‘I am ashamed that I am HIV positive’). Responses to these items included either ‘agree’ (1) or ‘disagree’ (0). Total score was obtained by summing the scores of seven items, with higher scores signifying a greater burden of internalized stigma.

In the multivariable analysis, those with lower levels of perceived family support had 6.05-fold higher odds of being ART eligible than those with higher levels of perceived family support (95% confidence interval=1.95–18.73; Table 2). A higher proportion of participants with secondary or higher levels of education (60%) reported significantly higher levels of perceived family support than those with lower educational levels (20.0%; *p*=0.003).

**Table 2:** Factors associated with antiretroviral therapy eligibility among HIV-positive individuals (*n*=72)

	Antiretroviral therapy eligibility^a^			
			
	Yes (*n=*36)	No (*n=*36)		
Variable	*n* (%)	*N* (%)	OR (95% CI)	AOR^b^ (95% CI)
Perceived family support^c^ (Median=24)				
High (25–30)	11 (31.4)	24 (68.6)		
Low (10–24)	25 (67.6)	12 (32.4)	4.54 (1.68–12.25)	6.05 (1.95–18.73)*
Age (in years)				
20–30	14 (42.5)	19 (57.6)		
31–47	22 (56.4)	17 (43.6)	1.75 (0.68–4.48)	
Sex				
Female	13 (52.0)	12 (48.0)		
Male	23 (48.9)	24 (51.1)	0.88 (0.33–2.33)	
Current marital status				
Single	13 (46.4)	15 (53.6)		
Married	23 (52.3)	21 (47.7)	1.26 (0.48–3.26)	
Education				
Up to primary	11 (55.0)	9 (45.0)		
Secondary or higher	25 (48.1)	27 (51.9)	0.75 (0.26–2.13)	
Employed				
No	9 (39.1)	14 (60.9)		
Yes	27 (55.1)	22 (44.9)	1.90 (0.69–5.23)	
Months since testing HIV positive				
1–53	18 (51.4)	17 (48.6)		
54+	18 (48.6)	19 (51.4)	0.89 (0.35–2.25)	
HIV disclosure to any family member^d^				
No	7 (63.6)	4 (51.7)		
Yes	29 (48.3)	31 (36.4)	0.53 (0.14–2.01)	
Illicit drug use, past 6 months				
No	29 (48.3)	31 (51.7)		
Yes	7 (58.3)	5 (41.7)	1.49 (0.42–5.24)	
Current smoker				
No	16 (51.6)	15 (48.4)		
Yes	20 (48.8)	21 (51.2)	0.89 (0.35–2.27)	
Alcohol use, past 30 days				
No	28 (49.1)	29 (50.9)		
Yes	8 (53.3)	7 (46.7)	1.18 (0.37–3.69)	
Depressive symptoms				
No (BDI-I<20)	27 (46.6)	31 (53.4)		
Yes (BDI-I≥20)	9 (64.3)	5 (35.7)	2.06 (0.61–6.92)	
HIV symptom burden				
Low (16–30)	21 (58.3)	15 (41.7)		
High (31–74)	15 (41.7)	21 (58.3)	0.51 (0.20–1.30)	0.37 (0.12–1.13)
History of any disease, past 12 months				
No	17 (63.0)	10 (37.0)		
Yes	19 (42.2)	26 (57.8)	0.43 (0.16–1.14)	0.44 (0.14–1.36)
Internalized stigma score				
Low (7–10)	13 (39.4)	20 (60.6)		
High (11–14)	23 (59.0)	16 (41.0)	2.21 (0.85–5.69)	1.76 (0.61–5.07)

AOR, adjusted odds ratio; BDI, Beck Depression Inventory; CI, confidence interval; OR, odds ratio.

a As suggested by the national antiretroviral therapy guidelines (16), HIV-positive individuals in the country are eligible for ART when their CD4+ cell count result is <350 cells/mm^3^. Delays in ART initiation was defined when participants did not initiate ART despite having CD4+ cell count result <350 cells/mm^3^.

b In the multivariable model, all variables that were associated with ART eligibility at *p*<0.20 in the bivariate analysis (perceived family support, HIV symptom burden, history of any disease in past 12 months, and internalized stigma) were included.

c Perceived family support was measured using the 10-item scale; items included 1) feeling of being shown love and caring by family, 2) feeling of having an important role in family, 3) Feeling of being involved in family decision making, 4) feeling of being able to share feelings with family, 5) feeling of basic needs being met in family, 6) feeling of being supported by family when sick, 7) feeling of being disliked by family, 8) feeling (emotionally) of distance from family member(s), 9) having been physically hurt by family member(s), and 10) feeling of being exploited (for household and farming) by family.

d One participant did not respond to this question.

* *p*=0.002

## Discussion

This study found that over four-fifths of the ART-naïve, HIV-positive individuals (all women and over three-fourths of men) had monitored their CD4+ cell count within the past 6 months, yet half of these individuals had not initiated ART despite being eligible per national ART guidelines (16). This finding has important implications for bringing ART-naïve, HIV-positive individuals into treatment, thereby improving the ART coverage in Nepal, even after the country decides to treat all ART-naïve individuals to follow WHO's new treatment guidelines (2). The failure to initiate ART among the ART-eligible participants in our study, for example, cannot be explained by a lack of knowledge about the participant's serostatus or a lack of access to services as all of these participants had their CD4+ cell count tested by the government laboratory and all were affiliated with NGOs working with HIV-positive individuals. Our results underscore the importance of strengthening the monitoring of attrition from pre-ART care to ART initiation, particularly among those who are ART eligible.

Being ART eligible was strongly associated with lower levels of perceived family support. In families with low levels of attachment, positive family interactions are not expected (25) and familial relationships can even be stressful. In such families, affected members may not share personal issues (25), including HIV diagnosis and ART initiation. It is possible that HIV-positive individuals in such families may postpone ART initiation, which may potentially be due to efforts to conceal their HIV status from their family members (26). Our results have important implications for designing and implementing ART initiation interventions for family members of treatment-naïve individuals. Involving families of HIV-positive individuals with high levels of perceived support might improve ART initiation. However, involvement of families for those individuals reporting low levels of perceived support might be more appropriate only with their prior consent. As highlighted by Poudel et al. (27), the focus of the intervention should be on building on the strengths in the former group while mitigating the harms in families in the latter group.

This study has some limitations. First, caution is advised in generalizing the study findings to the larger population of HIV-positive individuals in the country, as we recruited our study participants through a network of NGOs serving HIV-positive individuals. Specifically, our study findings may be applicable to HIV-positive individuals in networks of NGOs, as existing in other parts of the country as well as in other resource-limited countries in Asia. Second, as our measures of perceived family support and other potential confounders, but importantly not CD4+ cell count, are based on self-report, it is possible that the responses of the participants may have been distorted by a social desirability bias, regardless of our efforts to minimize such bias by assuring the participants about the confidentiality of their information. Finally, our study was conducted with a relatively small number of ART-naïve, HIV-positive individuals. It is possible that delays in ART initiation might be even greater among the HIV-positive individuals who are not connected in the networks of NGOs and are located in areas with more limited access to ART. A nationally representative study, therefore, is necessary to estimate the actual rate of delays in ART initiation and explore strategies to improve the coverage of ART in the country.

Despite such limitations, high rate of delays in ART initiation and the strong association of perceived family support with ART eligibility among HIV-positive individuals highlight the importance of reassessing strategies for bringing more ART-eligible, HIV-positive individuals into treatment in the Kathmandu Valley.

## Paper context

Studies have reported high rates of late antiretroviral therapy (ART) initiation and its association with early mortality after ART initiation among HIV-positive individuals in the clinical settings. Our community-based study revealed that half of the ART-naïve HIV-positive individuals do not initiate ART despite being ART eligible. Our results underscore the importance of strengthening the monitoring of attrition from pre-ART care to ART initiation, particularly among those who are ART eligible.

## References

[1] UNAIDS (2012). UNAIDS report on the global AIDS epidemic 2012.

[2] WHO (2015). Guideline on when to start antiretroviral therapy and on pre-exposure prophylaxis for HIV.

[3] UNAIDS (2014). Fast-track: ending the AIDS epidemic by 2030.

[4] WHO (2013). Global update on HIV treatment 2013: results, impact and opportunities: WHO report in partnership with UNICEF and UNAIDS.

[5] UNAIDS (2015). AIDS by the numbers 2015.

[6] Kiertiburanakul S, Boettiger D, Lee MP, Omar SF, Tanuma J, Ng OT (2014). Trends of CD4 cell count levels at the initiation of antiretroviral therapy over time and factors associated with late initiation of antiretroviral therapy among Asian HIV-positive patients. J Int AIDS Soc.

[7] Lahuerta M, Ue F, Hoffman S, Elul B, Kulkarni SG, Wu Y (2013). The problem of late ART initiation in sub-Saharan Africa: a transient aspect of scale-up or a long-term phenomenon?. J Health Care Poor Underserved.

[8] Nash D, Wu Y, Elul B, Hoos D, El Sadr W (2011). Program-level and contextual-level determinants of low-median CD4+ cell count in cohorts of persons initiating ART in eight sub-Saharan African countries. AIDS.

[9] Brinkhof MW, Boulle A, Weigel R, Messou E, Mathers C, Orrell C (2009). Mortality of HIV-infected patients starting antiretroviral therapy in sub-Saharan Africa: comparison with HIV-unrelated mortality. PLoS Med.

[10] Fujita M, Poudel KC, Do TN, Bui DD, Nguyen VK, Kato M (2012). A new analytical framework of ‘continuum of prevention and care’ to maximize HIV case detection and retention in care in Vietnam. BMC Health Serv Res.

[11] MOHP (2012). Nepal country progress report 2012: to contribute to global AIDS response progress report 2012.

[12] Poudel KC, Palmer PH, Jimba M, Mizoue T, Kobayashi J, Poudel-Tandukar K (2014). Coinfection with hepatitis C virus among HIV-positive people in the Kathmandu Valley, Nepal. J Int Assoc Provid AIDS Care.

[13] Poudel-Tandukar K, Bertone-Johnson ER, Palmer PH, Poudel KC (2014). C-reactive protein and depression in persons with human immunodeficiency virus infection. Brain Behav Immun.

[14] Poudel-Tandukar K, Poudel KC, Jimba M, Kobayashi J, Johnson CA, Palmer PH (2013). Serum 25-hydroxyvitamin d levels and C-reactive protein in persons with human immunodeficiency virus infection. AIDS Res Hum Retroviruses.

[15] Poudel KC, Bertone-Johnson ER, Poudel-Tandukar K (2016). Serum zinc concentration and C-reactive protein in individuals with human immunodeficiency virus infection: the positive living with HIV (POLH) Study. Biol Trace Elem Res.

[16] MOHP (2009). National antiretroviral therapy guidelines.

[17] Fujita M, Poudel KC, Green K, Wi T, Abeyewickreme I, Ghidinelli M (2015). HIV service delivery models towards ‘Zero AIDS-related Deaths’: a collaborative case study of 6 Asia and Pacific countries. BMC Health Serv Res.

[18] Poudel KC, Nakahara S, Poudel-Tandukar K, Yasuoka J, Jimba M (2009). Unsafe sexual behaviors among HIV-positive men in Kathmandu Valley, Nepal. AIDS Behav.

[19] Poudel KC, Okumura J, Sherchand JB, Jimba M, Murakami I, Wakai S (2003). Mumbai disease in far western Nepal: HIV infection and syphilis among male migrant-returnees and non-migrants. Trop Med Int Health.

[20] Poudel KC, Poudel-Tandukar K, Yasuoka J, Joshi AB, Jimba M (2010). Correlates of sharing injection equipment among male injecting drug users in Kathmandu, Nepal. Int J Drug Policy.

[21] Kohrt BA Political violence and mental health in Nepal: war in context, structural violence, and the erasure of history.

[22] Martin C, Poudel-Tandukar K, Poudel KC (2014). HIV Symptom burden and anemia among HIV-positive individuals: cross-sectional results of a community-based positive living with HIV (POLH) study in Nepal. PLoS One.

[23] Kohrt BA, Kunz RD, Koirala RN, Sharma VD, Nepal MK (2009). Validation of a Nepali version of the Beck Depression Inventory. Nep J Psychiatry.

[24] Katz MH (2011). Multivariable analysis: a practical guide for clinicians and public health researchers.

[25] McCubbin HI, Thompson AI, McCubbin MA (1996). Family assessment: resiliency, coping and adaptation: inventories for research and practice.

[26] Wouters E, Meulemans H, van Rensburg HC (2009). Slow to share: social capital and its role in public HIV disclosure among public sector ART patients in the Free State province of South Africa. AIDS Care.

[27] Poudel KC, Buchanan DR, Amiya RM, Poudel-Tandukar K (2015). Perceived family support and antiretroviral adherence in HIV-positive individuals: results from a community-based positive living with HIV study. Int Q Community Health Educ.

